# Expression of Concern: Copywriters’ preference evaluation on online copywriting course attributes during the COVID-19 pandemic

**DOI:** 10.1371/journal.pone.0358924

**Published:** 2026-09-23

**Authors:** The PLOS One Editors

The *PLOS One* Editors issue this Expression of Concern due to concerns about the article’s [1] compliance with the PLOS Authorship policy. Readers are advised to interpret the article [1] with caution.
