# Supplementary material for: Copywriters’ preference evaluation on online copywriting course attributes during the COVID-19 pandemic
Source: PLoS One. 2024 May 23;19(5):e0292467. doi: 10.1371/journal.pone.0292467 (PMC11115326; doi:10.1371/journal.pone.0292467)
Supplement: Attachment — Submitted filename: PLOS.docx [file pone.0292467.s001.docx]

1. Title seems too generic. It should be made catchy and informative. It’s a bit longer. It would be better to have 12-15 words. It can be shortened by removing the methodological concept A Conjoint Analysis Approach.

Abstract presents a line about context and background of the study which does not clarify the authors intention towards this study. Moreover, it is not clear about the methodology adopted in the study. The authors have presented “Conjoint Analysis” as the research design used in the study. But they have not clarified whether it was survey, or case study or others and if it was qualitative, quantitative or mixed. The sample size is 292 and sampling is purposive. I am doubt how this number is possible to be selected from purposive sampling.

2. This section annexed both the background and the literature review. The researchers have begun their writing with thematic ideas which is good in any academic writing. Moreover, the authors have clearly explained these aspects in this section:

® their hope to add to this body of knowledge

® identify gaps or shortcomings in this knowledge

® explain why the gaps selected for investigation are important/significant enough for investigation (the rationale and significance of the research

® outline how they carried out the investigation, together with an indication of the scope and parameters of the research

® justify why the gap(s) are significant enough to be investigated.

® present the theoretical bases of the study

Moreover, they have started and ended writing with other’s statements which is not taken good in academic writing. They have ended a paragraph as “who only write copy for their employer’s products, and freelance copywriters – who offer their copywriting services on the market independently (Team, 2021)”

They need to follow the pattern of their words+ review + their words.

3. In this section the authors have presented two headings together as **Methodology**
   **3.1 Participants which is not enjoyable in academic writing. They have engaged** 292 Filipino copywriting respondents selecting purposively which is in doubt how this mass can be selected purposively.

They have used Conjoint Analysis but fail to justify the need of this method in this study which they must do. Moreover, they should explain the methodological approach that Conjoint Analysis comes under.

**Results**

The authors have presented the results/findings of the study that are relevant to the research objectives. The results are presented in tables using statistical devices standard deviation and mean value.

They have used single word as the heading of the table which does not sound good. They have used as **Table 4.** Utilities. They should mention the whole phrase as the table heading.

**Discussion**

The results were discussed comparing and contrasting with some published literature.

They have clearly stated the implication and contribution of this study

Conclusion

Conclusion is the authors’ space where they put their own reflection from the overall results and discussion of the research. However, the authors have begun conclusion as The restrictions that were imposed in response to the COVID-19 pandemic led to the inevitable reliance on online courses (Khan et al., 2021; Prasetyo et al., 2021; Sobaih et al., 2021), which does not sound better.

It does not carry the worth of conclusion that an academic writing should do. The authors should put their reflection which they fail to reveal in conclusion section.

References

I am in doubt whether the authors have followed APA 6^th^ or 7^th^ ed.

I prefer them to use APA 7^th^ .
