# Supplementary material for: Copywriters’ preference evaluation on online copywriting course attributes during the COVID-19 pandemic
Source: PLoS One. 2024 May 23;19(5):e0292467. doi: 10.1371/journal.pone.0292467 (PMC11115326; doi:10.1371/journal.pone.0292467)
Supplement: Attachment — Submitted filename: Response to Reviewers.docx [file pone.0292467.s002.docx]

Reviewer #1: 1. Title seems too generic. It should be made catchy and informative. It’s a bit longer. It would be better to have 12-15 words. It can be shortened by removing the methodological concept A Conjoint Analysis Approach.

**Response:** Thank you for your constructive suggestion. We have addressed this concern by eliminating the methodological concept as suggested. The title is now 12 words long.

Abstract presents a line about context and background of the study which does not clarify the authors intention towards this study.

**Response:** Thank you for your constructive suggestion. In order to further clarify our intention, we have changed the opening line and appended the following statement:

“In recent years, online courses have become a popular training platform especially for copywriting courses. The demand for online courses increased during the COVID-19 pandemic, prompting the need to optimize the learning experience of an online course’s target audience.”

Moreover, it is not clear about the methodology adopted in the study. The authors have presented “Conjoint Analysis” as the research design used in the study. But they have not clarified whether it was survey, or case study or others and if it was qualitative, quantitative or mixed. The sample size is 292 and sampling is purposive. I am doubt how this number is possible to be selected from purposive sampling.

**Response:** Thank you for your constructive suggestion. This insight was deemed important. As such, we have indicated on the abstract the use of an online quantitative survey and included where we sourced the respondents.

2. This section annexed both the background and the literature review. The researchers have begun their writing with thematic ideas which is good in any academic writing. Moreover, the authors have clearly explained these aspects in this section:
® their hope to add to this body of knowledge
® identify gaps or shortcomings in this knowledge
® explain why the gaps selected for investigation are important/significant enough for investigation (the rationale and significance of the research
® outline how they carried out the investigation, together with an indication of the scope and parameters of the research
® justify why the gap(s) are significant enough to be investigated.
® present the theoretical bases of the study
Moreover, they have started and ended writing with other’s statements which is not taken good in academic writing. They have ended a paragraph as “who only write copy for their employer’s products, and freelance copywriters – who offer their copywriting services on the market independently (Team, 2021)”
They need to follow the pattern of their words+ review + their words.

**Response:** Thank you for your constructive suggestion. We have highlighted the type of copywriter targeted by the study by appending the following statement:

“In recent years, the 3 main types of copywriters are dominated by freelance copywriters due to the emergence of freelancing platforms such as Upwork and Freelancer.”

3. In this section the authors have presented two headings together as Methodology
3.1 Participants which is not enjoyable in academic writing.

**Response:** Thank you for your constructive suggestion. Upon careful review, we have deemed the first paragraph of 3.1 participants to be better suited as the paragraph that would set the tone for 3. Methodology. As such, we have moved the paragraph to the 3. Methodology section, leaving the 3.1 participants to focus solely on the respondent demographics and attributes.

They have engaged 292 Filipino copywriting respondents selecting purposively which is in doubt how this mass can be selected purposively.

**Response:** Thank you for your constructive suggestion. This insight was deemed important and as such, this issue has been addressed by disclosing the source of the respondents, which is an online group of composed of Filipino copywriters.
They have used Conjoint Analysis but fail to justify the need of this method in this study which they must do. Moreover, they should explain the methodological approach that Conjoint Analysis comes under.

**Response:** Thank you for your constructive suggestion. In the introduction, we have discussed that conjoint analysis is most appropriate due to the significant number of online copywriting course attributes that needed to be analyzed in lieu of Filipino consumer preferences. Aside from Conjoint analysis being a popular tool for product marketing analysis and consumer research, Tang’s 2020 study on online course preferences on Chinese consumers used Conjoint Analysis as well.

Results
The authors have presented the results/findings of the study that are relevant to the research objectives. The results are presented in tables using statistical devices standard deviation and mean value.
They have used single word as the heading of the table which does not sound good. They have used as Table 4. Utilities. They should mention the whole phrase as the table heading.

**Response:** Thank you for your constructive suggestion. This is correct. As such, we have amended Table 4 by changing the table heading to ‘Online Course Attribute Utilities’ which properly describes Table 4.

Discussion
The results were discussed comparing and contrasting with some published literature.
They have clearly stated the implication and contribution of this study

Conclusion
Conclusion is the authors’ space where they put their own reflection from the overall results and discussion of the research. However, the authors have begun conclusion as The restrictions that were imposed in response to the COVID-19 pandemic led to the inevitable reliance on online courses (Khan et al., 2021; Prasetyo et al., 2021; Sobaih et al., 2021), which does not sound better.
It does not carry the worth of conclusion that an academic writing should do. The authors should put their reflection which they fail to reveal in conclusion section.

**Response:** Thank you for your constructive suggestion. We have appended the following paragraph to the conclusion section:

“It appears that Filipino copywriters are strongly opinionated on their preferences when it comes to online copywriting courses as shown by the significant gaps between utility estimates. They place high importance on Course style, particularly on Video courses, which reveals their preferred learning process. Another insightful observation is how the payment method became the second most important attribute. For course creators, this information can also be leveraged to marketing their courses.“

References
I am in doubt whether the authors have followed APA 6th or 7th ed.
I prefer them to use APA 7th .

**Response:** Thank you for your constructive suggestion. We’ve changed the references to follow the APA 7^th^ edition’s style manual. It is alphabetical and has hanging indents. The following sequence was used: last name and initial of authors, ampersand instead of ‘and’, publication year in parentheses, publication and article title in sentence case, academic journal in italics, volume number and issue number, page range, and DOI if available.

Reviewer #2: I have the following observations, questions, and comments that may help to improve your work. The authors must modify the following points in great detail.

1. In the abstract, please include 2-3 special quantitative achievements from the findings of this study in the context of the environment by combining the research objectives and problems. Please limit your abstract to 250 words. Check spellings for many words that are misspelt or written in haste.

**Response:** Thank you for your constructive suggestion. We have rewritten the abstract to included 4 quantitative achievements by identifying the most important and least important attributes as well as the best and worst course attribute combinations. The abstract is now 246 words long and has been proofread with Grammarly for an overall score of 94. The rewritten abstract as follows:

“In recent years, online courses have become a popular training platform, especially for copywriting courses. The demand for online courses increased during the COVID-19 pandemic, prompting the need to optimize the learning experience of an online course’s target audience. This study aimed to determine the combination of online course attributes most preferred by Filipino copywriters such as course style, payment method, course delivery, module duration, and course type. 292 Filipino copywriters from a leading Philippine-based copywriting group voluntarily participated in this study and answered an online quantitative survey which was distributed using the purposive sampling method. Conjoint Analysis with an orthogonal design revealed that copywriters consider the course style attribute as the most important (46.007%), while the course type (9.833%) was the least considered attribute of an online course. The result shows that Filipino copywriters prefer an intermediate-level video course on a Facebook group that lasts 1 to 3 hours per module and is paid per course for a total utility score of 0.281, while the least preferred combination was a beginner-level audiobook course that lasts less than 30 minutes per module, delivered via email, and paid per module, for a total utility score of -0.281. This study is the first study that analyzed the copywriters' preference for online copywriting course attributes during the COVID-19 pandemic. The findings of this study are beneficial to online course creators who are targeting copywriters. Finally, the result of this study can be expanded further to other online courses worldwide.”

2. The introduction section needs a few more sentences to strengthen the article, and please include the research problem, objective, and novelty in the last paragraph of the Introduction section.

**Response:** Thank you for your constructive suggestion. In the last paragraph of the introduction section, we have placed the research problem to highlight the need to achieve the objective and then explained how our study is the first of its kind, especially in the Philippines and in the field of copywriting.

3. Include a few more sentences at the beginning of the introduction explaining your paper's contribution to online education, as well as your attempts to deal with or present solutions to a specific problem/s and your unique contribution with this research paper.

**Response:** Thank you for your constructive suggestion. We have included the following statement as follows:

“The insights gained from this study may serve as a foundation for improving online education by providing key course attributes that will help online courses become more appealing and more engaging, which will improve adoption and utilization especially during a pandemic.”

4. Please also present the methodology section in a concise graphical format.

**Response:** Thank you for your constructive suggestion.

5. The literature review section is very weak; please revise it.

**Response:** Thank you for your constructive suggestion.

6. Please present your literature review in the form of a SmartArt chart.

**Response:** Thank you for your constructive suggestion.

7. Just after the Methodology, please mention the societal benefits of your research in terms of evaluating its key determinant.

**Response:** Thank you for your constructive suggestion. We have appended the following statement:

“The insights to be gleaned from this analysis should reveal key attributes and attribute combinations to adopt and to avoid in order to improve the appeal, adoption, and engagement of online courses, especially during a pandemic where in-person interactions are limited.”

8. In 500-750 words, explain research problems, solutions, and the theoretical contribution of your study in the "Results" section.

**Response:** Thank you for your constructive suggestion. We have revised it accordingly.

9. Please include graphical presentations of your findings.

**Response:** While utilizing conjoint analysis, graphical presentations are not suitable. Instead, we presented in a summary table. We believe this summary table is more than enough to represent the findings of the study.

10. Describe why you placed this study in a separate section of "Policy Suggestions" just before the section of "Conclusions."

**Response:** We do not have a separate section of “Policy Suggestions”.

I found that the literature section is a little weak, shift your study a little more towards online learning technologies therefore it requires more studies to be reviewed therefore I suggest you to include the following work:

**Response:** Please send us the links since we cannot find the links in your comments.
