# Supplementary material for: Copywriters’ preference evaluation on online copywriting course attributes during the COVID-19 pandemic
Source: PLoS One. 2024 May 23;19(5):e0292467. doi: 10.1371/journal.pone.0292467 (PMC11115326; doi:10.1371/journal.pone.0292467)
Supplement: Attachment — Submitted filename: Response to Reviewers.docx [file pone.0292467.s003.docx]

Reviewer #3: Dear Authors,
Congratulations for your interesting research. I have some suggestions on how to make your text more attractive for wider audience.
1. Your topic should be embedded in a broader discussion of course style, payment methods, how the course is delivered, module duration and type of course in other countries. Suggest that there should be two or three examples in the article showing whether the example of the Philippines differs in the topic under discussion or whether there are similarities in other countries as well. the decreasing affordability of housing in different countries

Answer: **Thank you for pointing this out. In consideration of your recommendation, the following was added to the introduction:**

*Udemy, one of the most popular online course platforms with millions of students enrolled, reported a significant growth in enrollment of technical skills geared mainly towards AI and machine learning, and a significant growth in soft skills such as growth mindset, creativity, focus, innovation, and communication. Significant growth in enrollments were found in Spain, India, and Italy (Udemy Special Report 2020). In terms of topics enrolled in Udemy by geographic profiles, there’s a massive spike of US enrollees for creative skills like Adobe Illustrator, enrollees from Spain are taking courses in piano and investing, Indian enrollees are taking business fundamentals and communication skills courses, while students from Italy are taking courses on guitar, Photoshop, and copywriting (Ederle, 2020). Most Udemy courses are paid per course and delivered via video within the platform, complete with modules lasting from 15 minutes to an hour. Coursera, another popular online course platform mostly used by students and professionals for upskilling, reported in 2022 that Philippine-based learners focused mostly on business-related courses at 62%. The online course platform also stressed that skill development strengthens island nations such as Singapore, Indonesia, and Japan. While most of Coursera’s courses are paid per course, which lasts for up to 3 months, and are delivered via video within the platform, the platform also offers special subscriptions where students can pay monthly to access multiple courses.*

2. Some amendments in the format are due to admit publication. The main problem is the epistemological structure (why the article was conceived and how the study was developed). I suggest the following structure of objectives: (i) research gap; (ii) research question; (iii) purpose of the article; (iv) intermediate objectives ; (v) assumptions or hypo; and (vi) research method. This structure must appear in the introduction.

Answer: **Thank you for pointing this out. In consideration of your recommendation, the introduction was revised as such:**

*On adoption and effect, a Thailand-based study on non-English major Thai students’ approach to a 3-month-long synchronous online English course showed that anxiety is not a significant predictor of student performance and that there are no differences in performance between genders (Apridayani et al., 2023). While in Israel, Israeli creative arts therapies master’s degree students showed a deeper appreciation and commitment to using their knowledge and skills after taking an online course on palliative and bereavement care, which was delivered in the last four sessions of the program. The courses were delivered asynchronous and synchronous, where the latter is a 3-hour session delivered by Ph.D. level experts via Zoom (Orkibi et al., 2023). An interesting study on Chinese nursing students and online courses showed that students with low family incomes and those who live in rural areas are not readily accepting of fully immersive online learning, while older male nursing students are more likely to fully immerse themselves when learning online. (Zhang et al., 2022). On the other hand, both architecture students and faculty of a university in Jordan, have shown satisfaction with online teaching when it comes to theoretical courses and dissatisfaction over the design courses, which infers that the online course’s topic’s level of technical involvement may influence perceived satisfaction as well (Ibrahim et al., 2021). From a commercially-driven perspective, a 2020 study by Zhang et al. shows that paid online courses are primarily purchased based on ratings and followers, while the reverse is observed on upvotes. This is tangentially affirmed by another 2020 study on free and paid e-books, which shows that consumption of e-books were primarily driven by ranking regardless of whether the e-book is paid or free, while the number of reviews doesn’t affect readers’ intention (Liu et al., 2020). An in-depth study of consumers’ purchase intention on online paid knowledge in China has revealed that the most important purchase factor is the course creator’s professionalism, followed by the course platform’s information quality and interactivity, and then by the rarity of the information being sold and the course creator’s perceived charisma (Zhou et al., 2022). Based on what the proponents of the studies were able to find, there are currently no studies referring to and reporting on the paid online course environment of the Philippines and paid online copywriting courses.*

*Considering the unprecedented reliance on online courses caused by the COVID-19 pandemic and the need to create an effective online copywriting course suited to Filipino copywriter’s preferences in order to sustain livelihood and economy, there is a need to determine which online course attributes are the most preferred by Filipino copywriters in order to create a course that is appealingly packaged and pedagogically effective and subsequently streamline acquisition of knowledge and skills to generate income.*

*As such, the aim of this study was to determine the preferred online copywriting course attributes of Filipino copywriters of varying experience levels by uncovering the most preferred attribute, the most preferred attribute combination, the ordered attribute preferences, as well as the least preferred attribute and its combinations so that these attributes won’t be adopted in future courses.*

3. The research gap must be created by a systematic literature review that provides 'holes' in the state of knowledge on the topic. I believe that a full review should not be done, but an analysis of about 5-8 studies on the topic under discussion. You can find some examples, which will show the relevance of the issue, as it is indeed a topic of current, relevant research. At the end of the justification you should write something like: According to what we were able to find, there are no studies referring and reporting on ... With this you have therefore proven that the issue is relevant, and you have also proven that your study does indeed fill a research gap.

Answer: **Thank you for pointing this out. We have addressed this on #2.**

4. In conclusion, I propose:
-evaluate the critical research, show its limitations and weaknesses,
- highlight the new knowledge and the lessons learned from it,
- describe the importance of the research and how it affects the wider field, show how the information obtained can be further used.
Conclusions must be clearly and unambiguously linked to the results of the survey. Their theoretical and practical implications should be indicated

Answer: **Thank you for pointing this out. In consideration of your recommendation, the conclusion was revised as such:**

*The significant findings and contributions of this study must be considered within the context of some limitations. First, the data collection and preference measurement were made through an online survey that was deployed in a Facebook group during the COVID-19 pandemic. Considering that the Facebook group, Copywriting Dojo Philippines, is a beginner-friendly community and that the job security issues caused by the pandemic led to a surge of Facebook group members, which led to beginner-level copywriters becoming the major respondents. More experienced and advanced respondents may result in a finding that may be useful for creating strategies for retaining participants in online courses. Furthermore, the study is only focused on Filipinos who are copywriters. Future research could include data from other nationalities and other industries that will benefit from online courses as this would help provide a general course preference and become the online course standard. Moreover, clustering techniques such as K-Means may highlight also the target demographics to promote the online course. Lastly, the online course attributes that were evaluated in this study were based on online courses usually offered on Facebook groups in the Philippines. In future research, it would be more enlightening to include installment payments, course creator interactions, peer-to-peer interactions, assignments, projects, instructor reputation, and the inclusion of bonus content as part of the course attributes and levels to be considered.*

*It appears that Filipino copywriters are strongly opinionated on their preferences when it comes to online copywriting courses as shown by the significant gaps between utility estimates. The results of this study show that when it comes to online courses, Filipino copywriters primarily prefer watching videos, paying per course, and enjoying their course on a website. For online course creators targeting Filipino copywriters, this means they can reduce the complexity of their course-building decisions and speed up the production of their course. From a sales perspective, this also means that they can attract more students and generate more revenue with a course that matches their preferences. Competition-wise, the result of this study should lead to a healthy online course competition based on the quality of the instructor, the curriculum, and content, which will help a lot of Filipino copywriters gain and improve their copywriting skills in the most efficient way possible. On a global scale, this also means there will be an ample supply of copywriting talent to meet the unprecedented demand caused by the COVID-19 pandemic. Therefore, these attributes and findings could be capitalized on by entrepreneurs to have higher engagement in online courses offered to heighten their businesses.*

*These insights are important as the restrictions that were imposed in response to the COVID-19 pandemic led to the inevitable reliance on online courses [32, 46, 47], especially for aspiring Filipino copywriters who need to learn new skills to generate a new source of income and practicing Filipino copywriters who need to improve their skills to keep their jobs or take on more clients. This study utilized Conjoint Analysis with an orthogonal design to identify the online course attribute combination most preferred by Filipino copywriters. Packaging the survey through a Conjoint Analysis approach made it easier for the 292 Filipino copywriters who voluntarily participated in the online survey by limiting the selection to with a total of 29 online course combinations presented. Course style, payment method, course delivery, module duration, and course type were used as the online course attributes to be evaluated.*

*Overall, this study found that course style, or the course format, is significantly the most important attribute that Filipino copywriters want in an online course. It indicates that Filipino copywriters are inclined to first consider how they are going to consume the online course. The same perspective goes for the online course payment method and course delivery, which signals the need for a convenient way to access the course. Based on these findings, the proponent recommends that online course creators should prioritize their course-building process around these 3 primary attributes first in order to increase the probability of enrolment from Filipino copywriters, and more importantly, reduce the number of factors to consider when testing and optimizing their courses. With these findings, this study can be considered the first on determining the online course preferences of Filipino copywriters. The findings of this study will strongly benefit course creators, the Filipino copywriting community, and even academicians who are interested to extend the study to other nationalities and industries.*

Good luck!

Reviewer #4: The article takes up quite an interesting title in terms of online courses and the current fashion for this type of course. However, the article needs to be refined. In the introduction, the authors write: " An exhaustive search on known research databases shows that this study on Filipino consumer preferences on online courses is the first of its kind, especially in the Philippines and in the field of copywriting." I recommend removing this sentence because it is not clear. I recommend that the authors substantively provide in this section what is novelty in the work and what gap is filled in the work.

Answer: **Thank you for pointing this out. We have removed this particular line from this study.**

Point 3: The methodology is not clear. This point requires solid refinement because it is described too broadly and little is known about the survey itself and the way it is analyzed. I recommend that the authors describe the above-mentioned point more substantively, especially in relation to how the questionnaire was built, the rating scale used, and the method of selecting the research sample.

Answer: **Thank you for pointing this out. In consideration of your recommendation, the following was added to the methodology:**

*Preliminary interviews were conducted with 2 Philippine-based copywriting course creators and 10 Philippine-based copywriters who took copywriting courses, which led to the determination of online course attributes and their subsequent levels. A 7-point Likert scale, repurposed as a satisfaction scale, was used in this study where 1 is labelled as ‘Highly Dissatisfied’ and 7 as ‘Highly Satisfied’. Respondents were provided a list of online course attribute combinations, as a visual reference, prior to rating. A homogenous purposive sampling was the approach used to secure the target respondent profile. This was done by disseminating an online survey on ‘Copywriting Dojo Philippines’, the first and largest copywriting community in the Philippines, therefore ensuring survey respondents are Filipino copywriters. The survey was accessible to the intended participants from July 1 to July 31, 2021. There were a total of 292 Filipino copywriting respondents who voluntarily participated in the self-administered survey.*

In conclusion, the authors write: "This was followed by payment method, course delivery, module duration, and course type." I definitely require in this section that I develop conclusions from the conducted research. I recommend that the authors elaborate on what specific payment method they are talking about and how attractive it is according to the respondents. Similarly, with course delivery, what are the respondents' requirements regarding delivery as well as module duration and course type? This section is definitely the weakest part of the work. In this section, the authors should convince the reader of the importance of the research carried out. I also reconsider here referring to the achievements of the literature in relation to the research results obtained.

Answer: **Thank you for pointing this out. In consideration of your recommendation, the conclusion was revised as such:**

*The significant findings and contributions of this study must be considered within the context of some limitations. First, the data collection and preference measurement were made through an online survey that was deployed in a Facebook group during the COVID-19 pandemic. Considering that the Facebook group, Copywriting Dojo Philippines, is a beginner-friendly community and that the job security issues caused by the pandemic led to a surge of Facebook group members, which led to beginner-level copywriters becoming the major respondents. More experienced and advanced respondents may result in a finding that may be useful for creating strategies for retaining participants in online courses. Furthermore, the study is only focused on Filipinos who are copywriters. Future research could include data from other nationalities and other industries that will benefit from online courses as this would help provide a general course preference and become the online course standard. Moreover, clustering techniques such as K-Means may highlight also the target demographics to promote the online course. Lastly, the online course attributes that were evaluated in this study were based on online courses usually offered on Facebook groups in the Philippines. In future research, it would be more enlightening to include installment payments, course creator interactions, peer-to-peer interactions, assignments, projects, instructor reputation, and the inclusion of bonus content as part of the course attributes and levels to be considered.*

*It appears that Filipino copywriters are strongly opinionated on their preferences when it comes to online copywriting courses as shown by the significant gaps between utility estimates. The results of this study show that when it comes to online courses, Filipino copywriters primarily prefer watching videos, paying per course, and enjoying their course on a website. For online course creators targeting Filipino copywriters, this means they can reduce the complexity of their course-building decisions and speed up the production of their course. From a sales perspective, this also means that they can attract more students and generate more revenue with a course that matches their preferences. Competition-wise, the result of this study should lead to a healthy online course competition based on the quality of the instructor, the curriculum, and content, which will help a lot of Filipino copywriters gain and improve their copywriting skills in the most efficient way possible. On a global scale, this also means there will be an ample supply of copywriting talent to meet the unprecedented demand caused by the COVID-19 pandemic. Therefore, these attributes and findings could be capitalized on by entrepreneurs to have higher engagement in online courses offered to heighten their businesses.*

*These insights are important as the restrictions that were imposed in response to the COVID-19 pandemic led to the inevitable reliance on online courses [32, 46, 47], especially for aspiring Filipino copywriters who need to learn new skills to generate a new source of income and practicing Filipino copywriters who need to improve their skills to keep their jobs or take on more clients. This study utilized Conjoint Analysis with an orthogonal design to identify the online course attribute combination most preferred by Filipino copywriters. Packaging the survey through a Conjoint Analysis approach made it easier for the 292 Filipino copywriters who voluntarily participated in the online survey by limiting the selection to with a total of 29 online course combinations presented. Course style, payment method, course delivery, module duration, and course type were used as the online course attributes to be evaluated.*

*Overall, this study found that course style, or the course format, is significantly the most important attribute that Filipino copywriters want in an online course. It indicates that Filipino copywriters are inclined to first consider how they are going to consume the online course. The same perspective goes for the online course payment method and course delivery, which signals the need for a convenient way to access the course. Based on these findings, the proponent recommends that online course creators should prioritize their course-building process around these 3 primary attributes first in order to increase the probability of enrolment from Filipino copywriters, and more importantly, reduce the number of factors to consider when testing and optimizing their courses. With these findings, this study can be considered the first on determining the online course preferences of Filipino copywriters. The findings of this study will strongly benefit course creators, the Filipino copywriting community, and even academicians who are interested to extend the study to other nationalities and industries.*
